# Home Dialysis Transitions in Canada During the COVID-19 Pandemic: An Interrupted Time Series Analysis

**DOI:** 10.1016/j.xkme.2025.101207

**Published:** 2025-12-12

**Authors:** Davide Verrelli, Reid Whitlock, Thomas Ferguson, Claudio Rigatto, Nathan Nickel, Karthik Tennankore, Oksana Harasemiw, Ranveer Brar, Clara Bohm

**Affiliations:** 1 Max Rady College of Medicine, University of Manitoba, Winnipeg, MB, Canada; 2 Chronic Disease Innovation Centre, Seven Oaks General Hospital, Winnipeg, MB, Canada; 3 Manitoba Centre for Health Policy, University of Manitoba, Winnipeg, MB, Canada; 4 Department of Medicine, Dalhousie University, Halifax, NS, Canada

**Keywords:** COVID-19 pandemic, dialysis transitions, hemodialysis, home dialysis, interrupted time series, peritoneal dialysis

## Abstract

**Rationale & Objective:**

During the coronavirus disease 2019 (COVID-19) pandemic, nephrology societies recommended transition from facility-based hemodialysis to home dialysis to minimize risks associated with COVID-19 infection. We compared transition rates from facility-based hemodialysis to home dialysis and rates and reasons for transfers from home dialysis to facility-based hemodialysis before and during the pandemic in Canada.

**Study Design:**

Interrupted time-series analysis.

**Setting & Population:**

Using administrative data from the Canadian Organ Replacement Register, our cohort included 31,596 and 22,607 adults with any time receiving hemodialysis during the prepandemic and pandemic study periods, respectively.

**Exposure:**

Early pandemic (April 1, 2020-September 30, 2021) versus prepandemic (January 1, 2016-December 31, 2019).

**Outcomes:**

Monthly rates of transitions between facility-based hemodialysis and home dialysis as well as reasons for transfer from home to facility.

**Analytical Approach:**

Segmented linear regression and analysis of covariance.

**Results:**

During the early pandemic, transitions to home dialysis increased by 0.60 per 10,000 patients/month (95% CI, 0.08 to 1.11; *P* = 0.03), beyond the nonsignificant monthly prepandemic trend of 0.02 per 10,000 (95% CI, –0.10 to 0.13; *P* = 0.80). Monthly transfers from home dialysis to facility-based hemodialysis per 10,000 home dialysis patients also increased during the pandemic (6.91; 95% CI, 3.42 to 10.40; *P* < 0.001) versus the prepandemic period (–1.78; 95% CI, –4.31 to0.75; *P* = 0.20). The rate of increase in home-to-facility transfers during the pandemic was not significantly different than facility-to-home transfers (−0.10 transfers/month; 95% CI, –1.51 to 1.31; *P* = 0.89). More transfers to facility occurred for geographic/resource-related reasons during the pandemic versus prepandemic (5.8% vs 2.7%; *P* < 0.0001).

**Limitations:**

Inability to analyze change in trends by province and ecological bias.

**Conclusions:**

Transitions from facility-based hemodialysis to home dialysis increased, suggesting kidney care programs in Canada implemented recommendations intended to decrease COVID-19-related risks in this population. Reasons for the observed increase in transfers from home to facility during the pandemic are unclear.

## Plain-Language Summary

During the coronavirus disease 2019 (COVID-19) pandemic, individuals with kidney failure receiving dialysis faced high rates of COVID-19 transmission and severe infection. As a preventive measure, kidney care societies advised increased use of home dialysis. To assess the impact of this directive, we tracked monthly transitions from facility-based hemodialysis to home dialysis before and during the pandemic using data from a large Canadian dialysis database. We found a significant increase in these transitions during the first 18 months of the pandemic compared with the prepandemic period. There was also similar increase in the number of transfers from home to facility dialysis, but it is unclear if this was due to the excess transitions to home dialysis we observed or other pandemic-related factors.

Most individuals who have kidney failure and need life-sustaining kidney replacement therapy receive facility-based hemodialysis.^1^ These individuals are immunocompromised and have multiple comorbid conditions, which, during the coronavirus disease 2019 (COVID-19) pandemic, significantly increased their risks of severe infection, higher rates of hospital admission and longer length of stay for COVID-19 infection, and increased mortality.^2^, ^3^, ^4^, ^5^, ^6^, ^7^, ^8^ Moreover, they had higher rates of COVID-19 infection compared with the general population, likely because of increased exposure through frequent hospital visits.^3^

At the beginning of the pandemic, anticipating the adverse effects of COVID-19 outbreaks in facility-based hemodialysis units, many national nephrology organizations recommended that kidney health providers transition patients from facility-based hemodialysis to home modalities when possible.^9^, ^10^, ^11^, ^12^, ^13^

Home modalities have traditionally been used by healthier individuals requiring dialysis, who are able to self-manage and are perceived to have the lowest likelihood of transfer from home dialysis to facility-based dialysis for medical or social reasons. Although home modalities are cost-effective,^14^, ^15^, ^16^, ^17^ likely improve quality of life in patients who value specific quality of life domains,^18^ and are as safe and effective as facility-based hemodialysis,^15^^,^^19^^,^^20^ home dialysis incidence and prevalence rates have remained unchanged in Canada despite significant efforts to increase home dialysis use. It remains unclear whether the current population of individuals using home dialysis can be expanded.

To identify whether opportunities to increase home modality use exist, we explored whether the rates of individuals transitioning from facility-based hemodialysis to home dialysis changed after the onset of the COVID-19 pandemic. In addition, we compared rates and reasons for transfer from home dialysis to facility-based hemodialysis before and during the pandemic period.

## Materials and Methods

### Study Population and Data Sources

**Figure 1:**
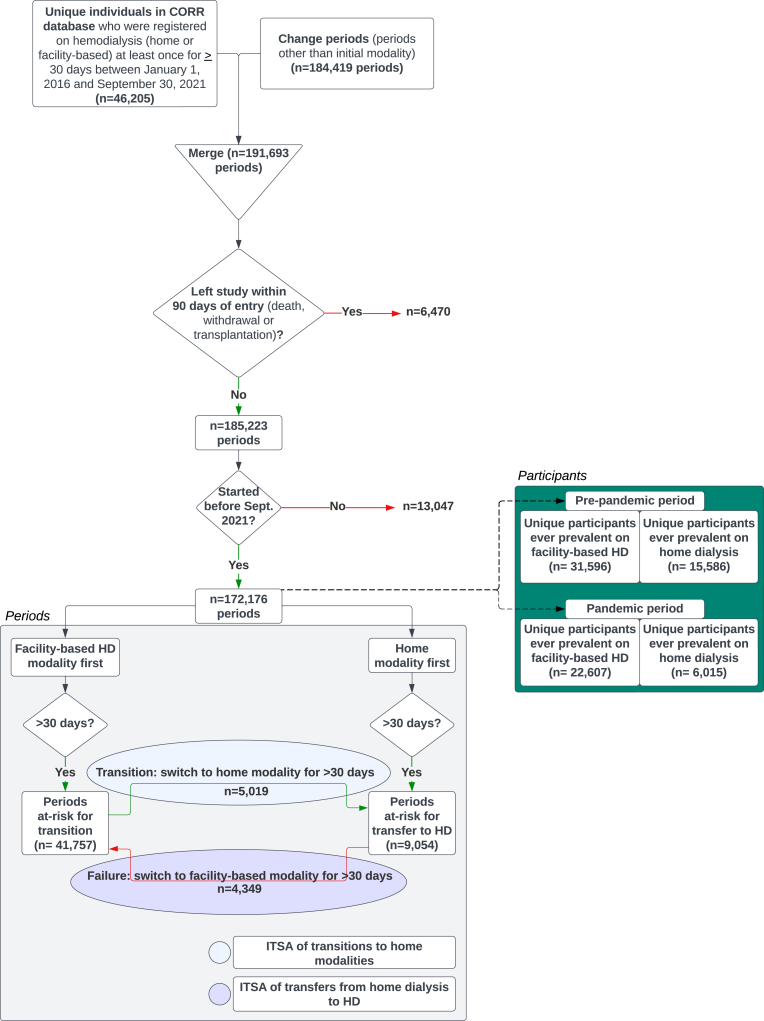
Study outline and cohort selection. Period is defined as a segment of time during which a participant was uniquely on either home dialysis or facility-based HD (minimum of 30 days). Some participants transitioned in both directions multiple times over the course of the study and thus were at risk of transition in either direction more than once. This figure demonstrates discrete periods and not the number of participants. Abbreviations: HD, facility-based hemodialysis; ITSA, interrupted time series analysis.

This retrospective interrupted time series analysis used data from the Canadian Organ Replacement Register (CORR) and included all adults (≥ 18 years of age) who were receiving hemodialysis (in facility or at home) at least once from January 1, 2016, to September 30, 2021. Accordingly, individuals who were exclusively on peritoneal dialysis (PD) and never switched to hemodialysis for the study duration were not included in the acquired dataset. Individuals who received a kidney transplant, recovered kidney function (suggesting acute kidney injury), withdrew from dialysis treatment, or died within the first 90 days of starting treatment were excluded from analyses (Fig 1).^21^ Individuals residing in Quebec were not collected or reported in the raw data from CORR because of data privacy laws restricting use of deidentified data without first-person consent. Because Quebec constitutes about 22% of the Canadian population, our data likely excludes a similar percentage of people with kidney failure, assuming rates are similar in Quebec to the rest of Canada.^22^

We defined January 1, 2016, to December 31, 2019, as the prepandemic period; January 1, 2020, to March 31, 2020, as a transition (“washout”) period, wherein pandemic-related changes were rapidly implemented; and April 1, 2020, to September 30, 2021, as the pandemic period, corresponding with significant increases in COVID-19 cases in Canada.^23^

CORR is a government-mandated and validated administrative database held at the Canadian Institute for Health Information that captures comprehensive individual-level data regarding people receiving kidney replacement therapy in Canada from treatment initiation until death.^24^ Annual data quality documentation from CORR reports no change in response rates during the study period (2016-2021).^25^ Because CORR is regularly updated, nonresponse of dialysis centers is addressed in a timely and complete manner by CORR staff, who work with the implicated centers to improve reporting.^25^ Strategies to achieve this include trending of incident dialysis patients and cross-checking of aggregate-level data sources with patient-level data.^25^

We followed the Strengthening the Reporting of Observational Studies in Epidemiology and the Reporting of Studies Conducted Using Observational Routinely Collected Data reporting guidelines for observational studies and studies using routinely collected data.^26^^,^^27^

### Demographics

Baseline characteristics were collected for each participant at the time of their dialysis initiation and included age range (5-year intervals), sex, race, body mass index, province/territory, distance to the nearest dialysis facility, socioeconomic quintile, and comorbid conditions.

Quintiles denoting socioeconomic status were received pregenerated in the CORR database, having been derived using the Postal Code Conversion File Plus product developed by Statistics Canada.^28^ This tool serves as a bridge between specific postal codes and demographic/socioeconomic information (population characteristics, income levels, educational attainment, and employment status collected at the level of the smallest geographic units used in census data known as “dissemination areas” consisting of ∼400-700 persons).^28^

### Outcomes

The primary outcome was the change in monthly rate of transitions from facility-based hemodialysis to home dialysis per 10,000 individuals receiving facility HD. A transition was defined as a switch to a home modality for a period of ≥30 days. The monthly rate of transitions was defined as the number of transitions within each month divided by the number of individuals receiving facility-based hemodialysis at the start of that month.^29^

Secondary outcomes included change in monthly rate of transfers from home dialysis to facility-based hemodialysis per 10,000 people receiving home dialysis, defined as switches for ≥30 days. The monthly rate of home-to-facility transfers was calculated as the number of transfers over each month divided by the number of individuals receiving home dialysis within our cohort that month. Trends in monthly facility-based hemodialysis initiation rates (first modality individuals received for ≥30 days) and mortality rates per 10,000 individuals prevalent receiving facility-based hemodialysis were both compared between the 2 study periods to help interpret whether potential changes in these outcomes may have influenced observed trends. The absolute number of facility-to-home and home-to-facility transfers were also compared with each other before and during the pandemic. Reasons for transfers from home to facility dialysis were defined using prespecified categories in the CORR database and were included as an exploratory outcome.^24^

Approval for this study was obtained from the University of Manitoba Health Research Ethics Board (HS25525 [H2022:181]).

### Statistical Analysis

Baseline characteristics for the study populations were presented as mean (standard deviation [SD]). Categorical outcomes were presented as proportions. Standardized differences were used to compare continuous baseline characteristics between the pre- and postpandemic cohorts, and categorical data were compared with χ^2^ test analysis.

For evaluation of facility-to-home transfers, home-to-facility transfers, and mortality receiving facility-based dialysis, simple linear regression models using the least squares method were specified with autoregressive errors applied as necessary. Initiations receiving facility-based hemodialysis were expressed as a count, so a generalized linear model with a natural log link and Poisson distribution was used. A negative binomial regression was conducted on the same data to assess for overdispersion, and the negative binomial dispersion parameter was zero, indicating there was no overdispersion.^30^ Our data were aggregated so that each month was a separate data point. The final model was structured as an interrupted time series analysis with proportions determined at monthly segments to calculate monthly rates.^31^ This model included a comparison of the trends in monthly rates (ie, slope) over the prepandemic period (January 1, 2016-December 31, 2019) versus the pandemic period (April 1, 2020-September 30, 2021). Home-to-facility transfers were evaluated using a prepandemic period restricted to July 1, 2018-December 31, 2019 to remove an artificial lag in completeness of the denominator introduced by the study inclusion criteria. Mortality rates on facility-based dialysis were evaluated as a proportion of individuals prevalent on facility-based dialysis each month, stated per 10,000 people. We used analysis of covariance via general linear modeling to compare the absolute number of home-to-facility and facility-to-home transfers during the prepandemic and pandemic periods.

The 2-sided 2-sample z test for proportions was used to compare changes in the proportion of reasons for home-to-facility transfers, defined as the count of a given reason divided by the total number of transfers in the period (prepandemic or pandemic). Cells containing <5 observations were suppressed to avoid potential identification of individuals. Cases with missing reasons for home-to-facility transfers were excluded in the final tabulations. All analyses were performed with SAS software, version 9.4 (SAS Institute, Inc).

Subgroup analyses were performed to distinguish whether the change in overall transition rates to home dialysis could be attributed to change in transition rates to home hemodialysis or PD and if the change in home-to-facility transfer rates could be attributed to changes in transfer rates specifically from home hemodialysis or PD.

## Results

In the final cohort, there were 31,596 prevalent individuals receiving facility-based hemodialysis during the prepandemic period and 22,607 prevalent individuals during the pandemic period, respectively (Fig 1). Compared with the prepandemic cohort, individuals in the pandemic cohort were more likely to be younger and non-White. Comorbid conditions were found to be not significantly different between the groups (Table 1).

**Table 1:** Characteristics of Prevalent Individuals Receiving Facility-Based Hemodialysis Between January 1, 2016, and September 30, 2021

Characteristics	January 1, 2016-December 31, 2019 (n = 31,596)	April 1, 2020-September 30, 2021 (n = 22,607)	Standardized Difference (d)	*P* Value
Age range (y)				<0.001
<30	741 (2.4)	583 (2.6)		
30-34	595 (1.9)	471 (2.1)		
35-39	780 (2.5)	569 (2.5)		
40-44	1,112 (3.5)	812 (3.6)		
45-49	1,625 (5.2)	1,236 (5.5)		
50-54	2,291 (7.3)	1,714 (7.6)		
55-59	3,034 (9.7)	2,325 (10.4)		
60-64	3,627 (11.6)	2,673 (11.9)		
65-69	4,346 (13.8)	3,124 (13.9)		
70-74	4,199 (13.4)	3,212 (14.3)		
75+	9,042 (28.8)	5,729 (25.5)		
Sex n (%)			0.074	
Female	12,549 (39.7)	9,088 (40.2)		
Male	19,047 (60.3)	13,519 (59.8)		
Race n (%)			0.088	
White	19,696 (62.3)	13,121 (58.0)		
Non-White	11,900 (37.7)	9,486 (42.0)		
Body mass index (kg/m^2^)	28.2 ± 6.7	28.4 ± 6.7	–0.027	
Province				0.11
British Columbia	4,661 (14.8)	3,423 (15.1)		
Alberta/Northwest Territories	3,452 (10.9)	2,608 (11.5)		
Saskatchewan	1,300 (4.1)	910 (4.0)		
Manitoba	2,263 (7.2)	1,659 (7.3)		
Ontario	16,803 (53.2)	11,804 (52.2)		
Atlantic Provinces	3,117 (9.9)	2,203 (9.7)		
Distance to facility (km)				<0.001
0-4	10,911 (34.9)	7,207 (32.2)		
5-9	7,076 (22.6)	5,082 (22.7)		
10-14	3,013 (9.6)	2,248 (10.1)		
15-19	1,768 (5.7)	1,302 (5.8)		
20-149	6,599 (21.1)	4,995 (22.3)		
150+	1,892 (6.1)	1,525 (6.8)		
Socioeconomic quintile				<0.001
First (lowest)	11,912 (40.1)	8,429 (39.9)		
Second	5,659 (19.0)	3,760 (17.8)		
Third	3,857 (13.0)	2,782 (13.2)		
Fourth	3,135 (10.5)	2,193 (10.4)		
Fifth (highest)	5,155 (17.3)	3,958 (18.7)		
Comorbid conditions				
Myocardial infarct	4,936 (16.5)	3,118 (14.4)	0.058	
Pulmonary edema	6,822 (23.0)	4,453 (20.8)	0.054	
Type 1 diabetes	244 (0.8)	175 (0.8)	<0.001	
Type 2 diabetes	4,553 (15.0)	3,451 (15.9)	–0.024	
Cerebrovascular disease	3,664 (12.2)	2,451 (11.3)	0.028	
Peripheral vascular disease	4,289 (14.3)	2,711 (12.5)	0.054	
Malignancy	3,872 (13.1)	2,615 (12.2)	0.028	
Lung disease	3,283 (11.0)	2,148 (9.9)	0.035	
Hypertension	24,776 (82.0)	18,315 (84.4)	–0.063	
Current smoker	4,813 (16.5)	3,492 (16.5)	0.001	
CABG	4,503 (15.0)	3,045 (14.0)	0.027	
OSI-5	4,074 (16.8)	2,900 (16.9)	−0.003	

Variables ascertained at the time of each participant’s dialysis initiation.

Data shown as n (%), mean ± SD and median(IQR).

Abbreviations: HTN, hypertension; OSI-5, other serious illness that could shorten life by 5 years.

Missing data for the following: age, n = 363 (0.007%); sex, n = 0; race, n = 0; body mass index, n = 0; province, n = 0; distance to facility, n = 585 (0.01%); socioeconomic quintile, n = 3,363 (0.06%); myocardial infarct, n = 2,484 (0.05%); pulmonary edema, n = 3,068 (0.06%); type 1 diabetes mellitus, n = 2,184 (0.04%); type 2 diabetes mellitus, n = 2,184 (0.04%); cerebrovascular disease, n = 2,395 (0.04%); peripheral vascular disease, n = 2,483 (0.05%); malignancy, n = 3,105 (0.06%); lung disease, n = 2,574 (0.05%); HTN medication, n = 2,282 (0.04%); osi-5, n = 12,876 (0.24%); current smoker, n = 3,908 (0.07%); coronary artery bypass, n = 2,416 (0.04%).

### Transitions From Facility-Based Hemodialysis to Home Dialysis

**Figure 2:**
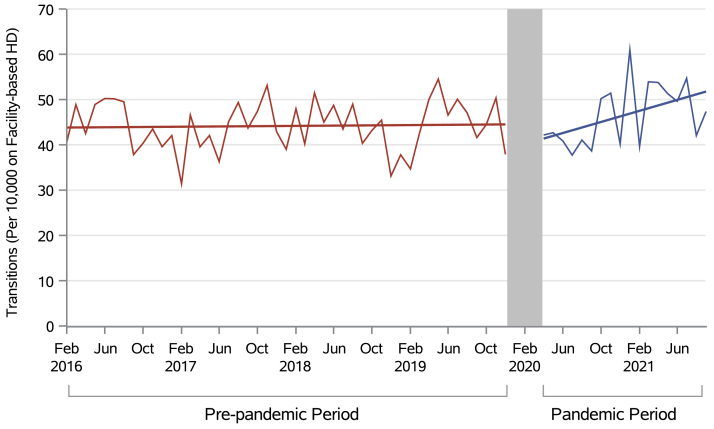
Transitions from facility-based hemodialysis to home dialysis in Canada over time per 10,000 monthly prevalent individuals receiving facility-based hemodialysis.

Before the pandemic, there were 0.0002% more transitions each month compared with the last (an extra 0.02 per 10,000 receiving facility dialysis each month; 95% CI, –0.10 to 0.13; *P* = 0.80), and the finding was nonsignificant. There was no significant immediate change in the rate of transitions at the onset of the pandemic (–3.79 per 10,000; 95% CI, –10.20 to 2.66; *P* = 0.30). We observed a significant increase in the rate of transitions to home dialysis during the pandemic by 0.006% per month (an extra 0.60 per 10,000 receiving facility dialysis each month; 95% CI, 0.08 to 1.11; *P* = 0.03), beyond the established prepandemic trend (Table 2, Fig 2).

**Table 2:** Results of Segmented Regression Models

Outcome	Segmented Regression Model Parameter	Coefficient (95% CI)	*P* Value
Transitions from facility-based hemodialysis to home dialysis (per 10,000 receiving facility-based hemodialysis)	Trend before pandemic	0.02 (–0.10 to 0.13)	0.8
	Immediate consequence after pandemic onset and 3 month washout	–3.79 (–10.20 to 2.66)	0.3
	Change in trend between prepandemic and pandemic periods	0.60 (0.08-1.11)	0.03
Transfers from home dialysis to facility-based dialysis (per 10,000 receiving home dialysis)	Trend before pandemic	–1.78 (–4.31 to 0.75)	0.2
	Immediate consequence after pandemic onset and 3 month washout	–3.57 (–46.50 to 39.40)	0.9
	Change in trend between prepandemic and pandemic periods	6.91 (3.42-10.40)	<0.001
Initiations on facility-based hemodialysis (% change per month)^a^	Trend before pandemic	0.27 (0.16-0.38)	<0.0001
	Immediate consequence after pandemic onset and 3 month washout	–2.96 (–8.61 to 3.05)	0.3
	Change in trend between prepandemic and pandemic periods	–0.39 (–0.89 to 0.11)	0.1
Mortality rate among individuals receiving facility-based hemodialysis (per 10,000)	Trend before pandemic	–0.05 (–0.22 to 0.11)	0.5
	Immediate consequence after pandemic onset and 3 month washout	8.3 (–2.26 to 18.80)	0.1
	Change in trend between prepandemic and pandemic periods	0.19 (–0.67 to 1.05)	0.7

a See Table 3 for month-to-month changes expressed as incidence rate ratios.

### Transfers From Home Dialysis to Hemodialysis

**Figure 3:**
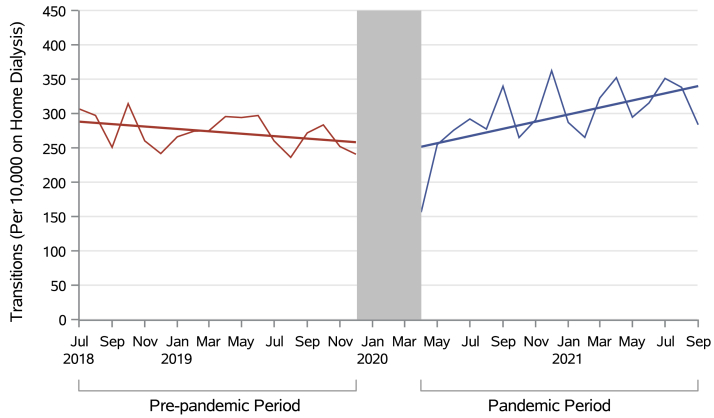
Transitions from home dialysis to facility-based hemodialysis in Canada over time per 10,000 monthly prevalent individuals receiving home dialysis.

Before the pandemic, there were 0.00178% fewer transfers each month compared with the last (or 1.78 fewer per 10,000 receiving home dialysis each month; 95% CI, –4.31 to 0.75; *P* = 0.20), which was nonsignificant. There was no significant immediate change at the start of the pandemic (3.57 per 10,000; 95% CI, –46.50 to 39.40; *P* = 0.90). During the pandemic, a significant increase in transfers to facility-based hemodialysis by 0.0691% per month (an extra 6.91 per 10,000 receiving home dialysis each month; 95% CI, 3.42 to 10.40; *P* = 0.0005) occurred, beyond the prepandemic trend (Table 2, Fig 3).

### Facility-Based Hemodialysis Initiations

**Figure 4:**
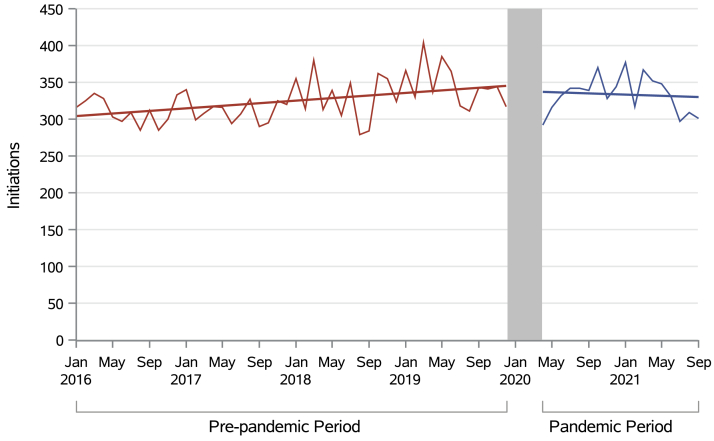
Absolute number of individuals initiating facility-based hemodialysis in Canada over time.

We observed an increasing trend in the rate of initiations receiving facility-based hemodialysis before the pandemic (incidence rate ratio [IRR], 1.0027; 95% CI, 1.0016 to 1.0038; *P* < 0.0001), meaning that each month the number of new patients starting facility-based hemodialysis increased by about 0.27%. There was no statistically significant change to this trend immediately after the onset of the pandemic (IRR: 0.9704; 95% CI: 0.9139, 1.0305; *P* = 0.30), or during the pandemic period (IRR, 1.0039; 95% CI: 0.9911, 1.0111; *P* = 0.10) meaning the prepandemic trend of increasing monthly initiations was conserved during the pandemic (Table 2, Table 3; Fig 4).

**Table 3:** Incidence Rate Ratios of Monthly Initiations Receiving Facility-Based Hemodialysis

Outcome	Segmented Regression Model Parameter	Incidence Rate Ratio	*P* Value
Initiations on facility-based hemodialysis	Trend before pandemic	1.0027 (1.0016-1.0038)	<0.0001
	Immediate consequence after pandemic onset and 3 month washout	0.9704 (0.9139-1.0305)	0.3
	Change in trend between prepandemic and pandemic periods	1.0039 (0.9911-1.0111)	0.1

### Facility-Based Hemodialysis Mortality Rates

**Figure 5:**
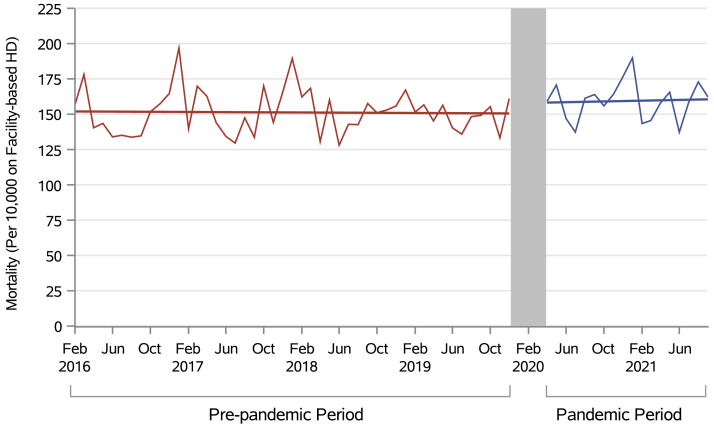
Mortality among patients receiving facility-based hemodialysis in Canada over time per 10,000 monthly prevalent patients receiving facility-based hemodialysis.

Before the pandemic, mortality among patients receiving facility-based hemodialysis remained stable, with a nonsignificant monthly increase of 0.0005% (0.05 deaths per 10,000 receiving facility-based hemodialysis; 95% CI, –0.22 to 0.11; *P* = 0.50). Immediately after the onset of the pandemic, there was a nonsignificant increase in deaths by 8.27 per 10,000 beyond the prepandemic trend (95% CI, –2.26, 18.80; *P* = 0.10). Finally, there was a nonsignificant increase in mortality of 0.19 deaths per 10,000 during the pandemic period beyond the prepandemic trend (95% CI, –0.67 to 1.05; *P* = 0.70) (Table 2, Fig 5).

### Differences in Home-to-Facility and Facility-to-Home Transfers

At the start of the prepandemic period, there was no significant difference between home-to-facility and facility-to-home transfers (8.42 more home-to-facility transfers/month; 95% CI, −2.6 to 19.51; *P* = 0.15), nor were the slopes during this period significantly different (0.858 fewer home-to-facility transfers/month; 95% CI, −1.88 to 0.17; *P* = 0.11). There was no significant difference at the start of the pandemic between average home-to-facility and facility-to-home transfers (3.37 more home-to-facility transfers/month; 95% CI, −40.28 to 47.02; *P* = 0.88), nor were the rates of increase in the 2 directions significantly different from each other during the pandemic (0.10 fewer home-to-facility transfers /month; 95% CI, –1.51 to 1.31; *P* = 0.89).

### Reasons for Transfers From Home Dialysis to Facility-Based Hemodialysis

A higher proportion of transfers to facility-based hemodialysis because of resource/geographical reasons occurred during the pandemic period (5.8%) compared with the prepandemic period (2.7%, *P* < 0.0001). Between the 2 study periods, there were no significant differences between proportions of transfers from home dialysis to facility dialysis because of peritonitis, other abdominal complications, other complications related to PD, home hemodialysis-related reasons, inadequate dialysis, transfer to originally intended treatment, dialysis recipient/family being unable to cope with the treatment, leaving the country, or “other” reasons (Table 4).

**Table 4:** Reasons for Transfer from Home Modality to Facility-based Hemodialysis Before and After April 1, 2020

Reason	July 1, 2018-December 31, 2019 (Total n = 1,294)	April 1, 2020-September 30, 2021 (Total n = 1,437)	*P* Value^a^
	N (%)	N (%)	
**Peritoneal dialysis related**	**259 (20.0)**	**279 (19.4)**	0.7
Peritonitis	95 (7.3)	132 (9.2)	0.08
Other abdominal complications	79 (6.1)	72 (5.0)	0.2
Other complications related to PD	85 (6.6)	75 (5.2)	0.1
**Home hemodialysis related**	**<10**	**<10**	0.6
Hemodialysis access failure	Supp.	Supp.	0.3
Cardiovascular instability	Supp.	Supp.	0.7
**Miscellaneous**			
Inadequate dialysis	68 (5.3)	82 (5.7)	0.6
Transferred to originally intended treatment	105 (8.1)	126 (8.8)	0.6
Dialysis recipient/family unable to cope with current treatment	76 (5.9)	62 (4.3)	0.06
Resource/geographical	35 (2.7)	84 (5.8)	<0.001
Left country	Supp.	Supp.	0.5
“Other” reason	726 (56.1)	779 (54.2)	0.3

Number of missing reasons for transfers: prepandemic period = 17; pandemic period = 19.

% = number of transfer from home dialysis to facility-based hemodialysis for given reason in period/total transfers in period.

Bolded rows denote aggregate (summary) totals, with subsequent rows representing the contributing components.

Abbreviation: Supp., suppressed for privacy because of insufficient cell size.

Mean number of participants at risk per month: prepandemic period = 2,675.9; pandemic period = 2,713.8.

a As calculated from 2 sample z test for proportions (2-sided).

### Subgroup Analyses Results

The monthly proportions of individuals transitioning from facility-based hemodialysis separately to either PD or home hemodialysis did not immediately change at the onset nor over the course of the COVID-19 pandemic period (Table 5; Figs S1, S2).

**Table 5:** Results From Segmented Regression Subgroup Analyses

Outcome	Segmented Regression Model Parameter	Coefficient (95% CI)	*P* Value
Transitions from facility-based hemodialysis to peritoneal dialysis	Trend before pandemic	0.02 (–0.08 to 0.11)	0.7
	Immediate consequence after pandemic onset and 3 month washout	–3.56 (–8.77 to 1.65)	0.2
	Change in trend between prepandemic and pandemic periods	0.28 (–0.14 to 0.70)	0.2
Transitions from facility-based hemodialysis to home hemodialysis	Trend before pandemic	–0.06 (–0.14 to 0.03)	0.9
	Immediate consequence after pandemic onset and 3 month washout	0.20 (–4.17 to 4.57)	0.9
	Change in trend between prepandemic and pandemic periods	0.29 (–0.06 to 0.64)	0.1
Transfers from peritoneal dialysis to facility-based hemodialysis	Trend before pandemic	–1.44 (–5.26 to 2.38)	0.5
	Immediate consequence after pandemic onset and 3 month washout	22.8 (–43.6 to 89.2)	0.5
	Change in trend between prepandemic and pandemic periods	6.20 (1.10-11.30)	0.03
Transfers from home hemodialysis to facility-based hemodialysis	Trend before pandemic	–2.68 (5.46-0.10)	0.07
	Immediate consequence after pandemic onset and 3 month washout	–36.7 (–83.9 to 10.5)	0.1
	Change in trend between prepandemic and pandemic periods	9.12 (5.32-12.90)	<0.001

Despite a stable prepandemic trend, there was a statistically significant increase in transfers from PD to facility-based hemodialysis of 0.062% per month (6.20 per 10,000 people; 95% CI, 1.10 to 11.30; *P* = 0.03) and transfers from home hemodialysis to facility-based hemodialysis HD (9.12 per 10,000; 95% CI, 5.32 to 12.90; P < 0.001) over the pandemic (Table 5; Figs S3, S4).

## Discussion

In this retrospective time series analysis, we observed a stable rate of transition from facility-based hemodialysis to home dialysis before the COVID-19 pandemic in Canada. Conversely, in the first 18 months of the pandemic, there was an overall increasing trend. This suggests that pandemic-related recommendations to transition individuals receiving facility-based hemodialysis to home dialysis to mitigate COVID-19 related risks were implemented in kidney care programs in Canada. Whether this shift to home modalities was solely due to these recommendations is uncertain, but timing of this change suggests that onset of the COVID-19 pandemic was a significant factor.

Although we were unable to identify any other national studies examining transitions from facility-based dialysis to home dialysis in the literature, the United States Renal Data Collecting System examines home dialysis use at baseline and 1 year among cohorts of patients with kidney failure onset on a yearly basis. In 2021, 21.0% of individuals with incident kidney failure were using home dialysis 1 year after onset—representing a nearly 60% relative increase compared with 2020, when 13.2% of patients had initiated home dialysis within 1 year of kidney failure onset.^32^ Although home dialysis initiation within the first year of kidney failure onset has been steadily growing in the United States, this increase during the pandemic in 2021 is the largest observed over the last decade, and the growth has been attributed to transitions from facility-based hemodialysis.^32^ Moreover, the increase in the number of prevalent patients receiving home dialysis in the United States slowed between 2020 and 2021, likely reflecting the increased mortality in the overall dialysis population over this time (159.1 deaths per 1,000 person-years in 2019 vs 186.4 in 2020).^32^

Importantly, we did not observe a statistically significant change in mortality rate in the facility-based dialysis population during the pandemic in Canada. Thus, the observed increase in monthly rate of transitions from facility-based hemodialysis to home dialysis was unlikely to be due to an increase in mortality rate and a consequent decrease in the denominator of prevalent individuals receiving facility-based dialysis over this time.

We also observed an increase in transfers from home to facility-based dialysis during the pandemic period. One possible explanation could be that more individuals who were marginal candidates for home dialysis were accepted for home modality training during the pandemic period, leading to accelerated technique failure and transitions back to facility-based hemodialysis. Alternatively, delays in seeking medical care, which were commonly observed during the COVID-19 pandemic,^33^ could have critically delayed treatment of medical or technical problems in the prevalent home dialysis cohort, leading to accelerated need for transfer to facility-based hemodialysis.^34^, ^35^, ^36^, ^37^ Indeed, across 136 hospitals surveyed in Mexico, 65 (47.8%) reported cancellation or postponement of consultations and procedures in PD centers, and 49 (36.0%) reported patient nonattendance because of fear of COVID-19 contagion during the first year of the pandemic.^38^

The increase in home-to-facility transfers is inflated by ∼55%-70% compared with estimates made with yearly CORR home dialysis prevalence rates because of our acquired dataset’s exclusion of individuals exclusively on PD (Table S1).^39^ Comparing 2019 to 2021, yearly home-to-facility transfers increased by 10/10,000 individuals prevalent receiving home dialysis (CORR’s complete denominator; Table S1), and facility-to-home transfers increased by ∼10/10,000 individuals prevalent receiving facility-based hemodialysis during this period (Fig 2). Our finding of a parallel increase in absolute number of transfers in both directions further corroborates that facility-to-home and home-to-facility transfers increased by the same magnitude during the pandemic.

We observed an increasing trend in facility-based hemodialysis initiations prepandemic, and this trend did not change during the pandemic. This contrasts with the observed decline in hemodialysis initiations across Europe and the United States in 2020, wherein the number of individuals starting hemodialysis decreased to levels not observed since 2013 and 2011, respectively.^32^^,^^40^

The overall incidence of kidney replacement therapy, including transplants and all dialysis initiations, in 2020 compared with the average of the prior 3 years also decreased in Europe (6.2%) and the United States (0.7%), whereas it increased in Canada (4.4%; CORR data).^1^^,^^41^ The smaller decrease in the United States compared with Europe was due in part to the increased incidence of peritoneal dialysis in the United States.^38^ Similarly, a 17.6% increase in the incidence of PD in Canada in 2020 compared with 2019 explains the bulk of the increase seen in overall initiations of kidney replacement therapy.^1^

In our exploratory analysis, resource- and geographical-related transfers were the sole reason for home-to-facility transfers that significantly changed during the pandemic period, demonstrating a doubling in frequency. In the CORR database, “resource/geographical” reasons for home-to-facility transfers include home dialysis unit staffing issues, equipment issues, and a lack of physical space in the home for dialysis machines and supplies (F. Ivis, CORR senior analyst, personal communication, July 28, 2023). Beyond these parameters, stressed supply chains during the pandemic could have resulted in disruption to the timely delivery of dialysis supplies, as has been corroborated in North America and virtually all other regions of the world,^38^^,^^42^ which could have affected people’s ability to continue receiving home modalities.

Minimizing caregiver and patient burnout may help mitigate transfers from home dialysis. Although populations receiving home dialysis have historically been younger, less frail, and healthier, assisted home dialysis, in which nurses or caregivers are formally trained and assist in home-based modalities, has been emerging as a way to broaden the eligible population for home dialysis.^43^ Local assisted home dialysis sites, in which individuals find sufficient storage space for dialysis supplies, adequate running water, and a nurse to help with select parts of treatment as needed have demonstrated value in Australia, New Zealand, and British Columbia, minimizing the unique challenges of dialysis in rural and remote areas and the costs associated with facility-based hemodialysis.^44^^,^^45^

Our study has several strengths. As the first inquiry into the impact of the COVID-19 pandemic on transitions from facility-based hemodialysis to home dialysis, this study provides important information to help guide clinical practice and care in future pandemics. Other strengths include the use of CORR data, a large administrative national database that has been previously validated,^46^ and the application of interrupted time series methodology, which allowed us to identify and control for prepandemic trends.

This study also has limitations. Our use of aggregated data to infer individual behavior may introduce ecological bias because such data can obscure confounders and within-group variability. Second, we could not subanalyze change in rate trends by province as low monthly outcome counts would have resulted in insufficiently powered analyses. Because of exclusion of Quebec data, study findings may not generalize to that province. Moreover, our cohort did not include individuals who were exclusively on PD, so monthly home-to-facility transfers could not accurately be stated as proportions of the complete home dialysis population. Finally, our results may not be generalizable outside of a universal, publicly funded health care setting.

Importantly, our finding that it is possible to increase transitions from facility-based hemodialysis to home modalities at a national level has public health, clinical, and research implications. First, the increase in transitions to home modalities occurring during a pandemic demonstrates that modality changes can be carried out promptly in response to public health crises, setting an example that can be followed for future public health crises. Considering ever increasing rates of kidney failure and capacity pressures within facility-based hemodialysis units in Canada and internationally, decision makers and clinicians should review program-level practices implemented during the pandemic to assess how modifying home dialysis eligibility criteria and increasing program supports can facilitate an ongoing increase in use of home modalities.^1^^,^^22^ Care should likewise be taken at the provincial level to better understand what factors led to the increase in home-to-facility transfers and to ensure only appropriate candidates are sent into the community for dialysis. Finally, mixed methods research examining reasons for resource-related home-to-facility transfers and developing support strategies to prevent these transfers may identify opportunities to extend the duration that individuals can continue to receive home dialysis.

In conclusion, we observed an increase in transitions from facility-based hemodialysis to home dialysis in Canada during the first 18 months of the COVID-19 pandemic as compared with the 4 years preceding the pandemic, suggesting that kidney care programs in Canada implemented recommendations intended to decrease COVID-19-related risks in this population. An equivalent increase in transfers from home dialysis to facility-based hemodialysis also occurred during the pandemic period; however, whether this increase was due to the excess transitions from facility-based hemodialysis or other pandemic-related factors is unclear.
