# Supplementary material for: Home Dialysis Transitions in Canada During the COVID-19 Pandemic: An Interrupted Time Series Analysis
Source: Kidney Med. 2025 Dec 12;8(2):101207. doi: 10.1016/j.xkme.2025.101207 (PMC12856449; doi:10.1016/j.xkme.2025.101207)
Supplement: Supplementary File (PDF) — Figures S1-S4; Table S1. [file mmc1.pdf]

Supplement Index

Supplemental Figures *2*

Figure S1. Transitions from facility-based hemodialysis to peritoneal dialysis in Canada over time per 10,000 monthly prevalent individuals on facility-based hemodialysis. 2

Figure S2. Transitions from facility-based hemodialysis to home hemodialysis in Canada over time per 10,000 monthly prevalent individuals on facility-based hemodialysis. 3

Figure S3. Transitions from peritoneal dialysis to facility-based hemodialysis in Canada over time per 10,000 monthly prevalent individuals on peritoneal dialysis 3

Figure S4. Transitions from home hemodialysis to facility-based hemodialysis in Canada over time per 10,000 monthly prevalent individuals on home hemodialysis. 4

Supplemental Table *5*

Table S1. Comparison of average monthly transfers to facility over a year per 10,000 individuals on home dialysis when not including versus including individuals prevalent on PD for the study duration (2016-2021) 5

Supplement

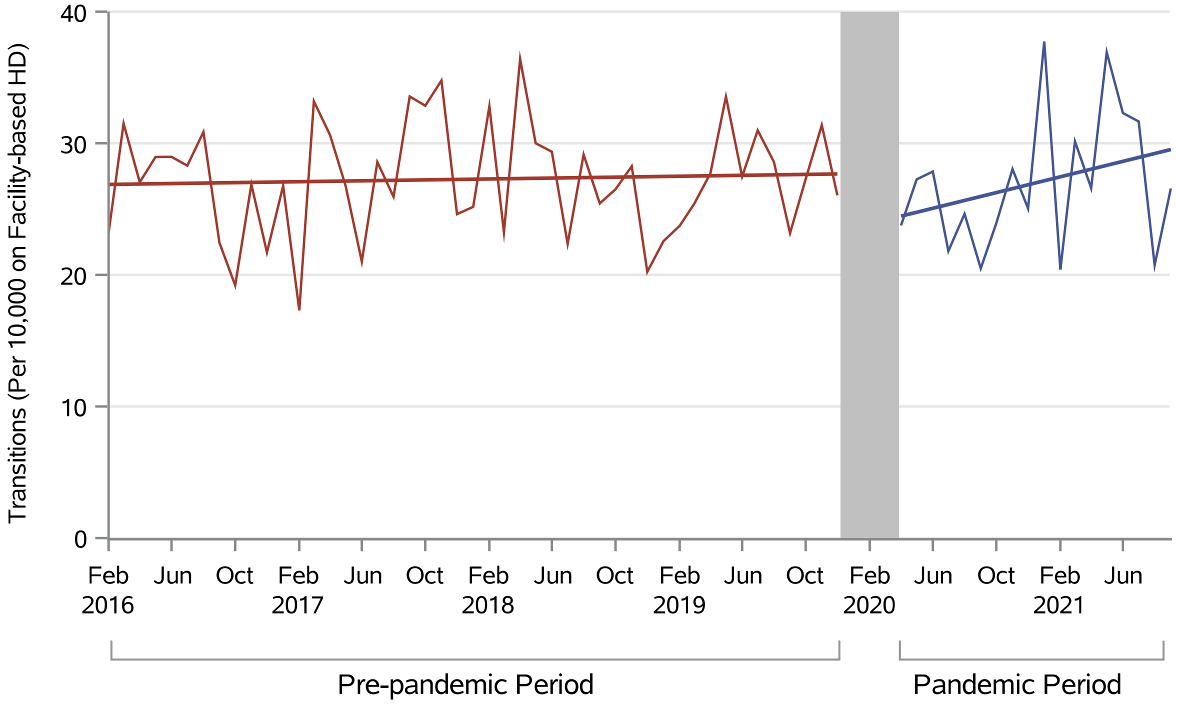

Supplemental Figures

Figure S1. Transitions from facility-based hemodialysis to peritoneal dialysis in Canada over time per 10,000 monthly prevalent individuals on facility-based hemodialysis.

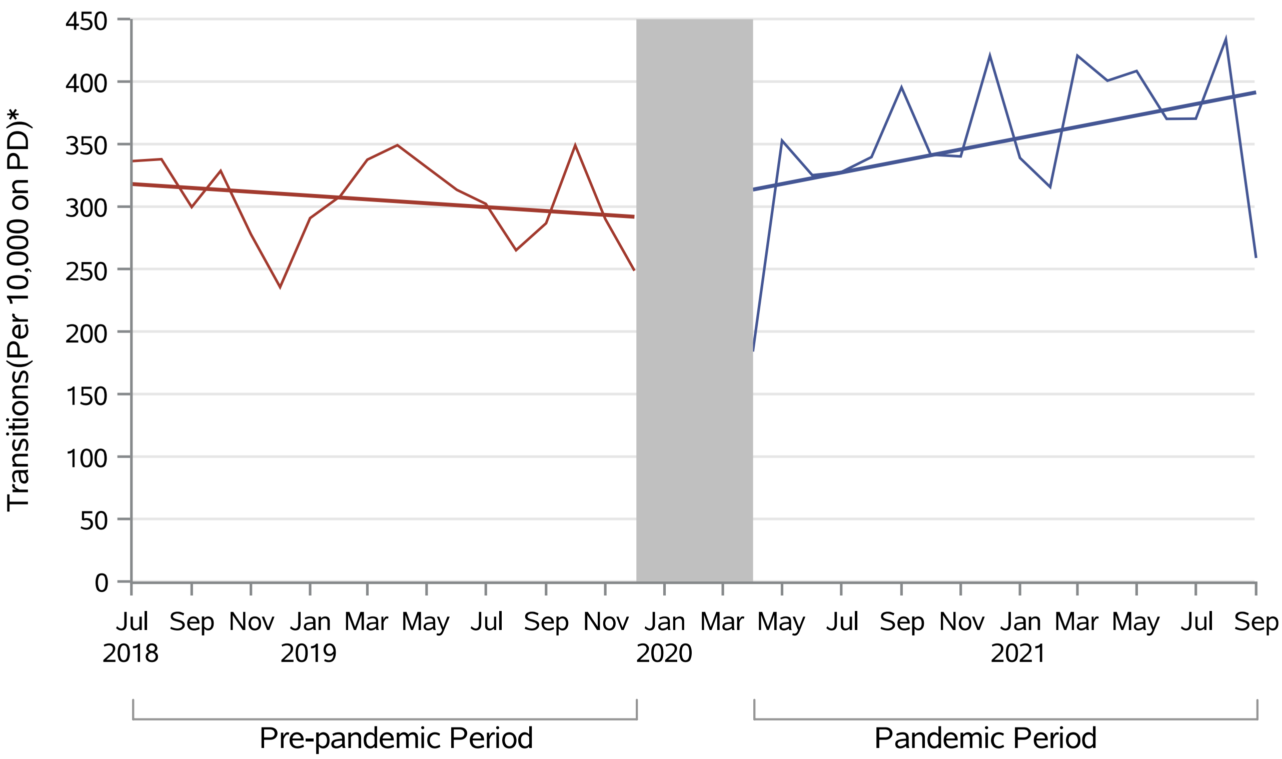

Figure S2. Transitions from facility-based hemodialysis to home hemodialysis in Canada over

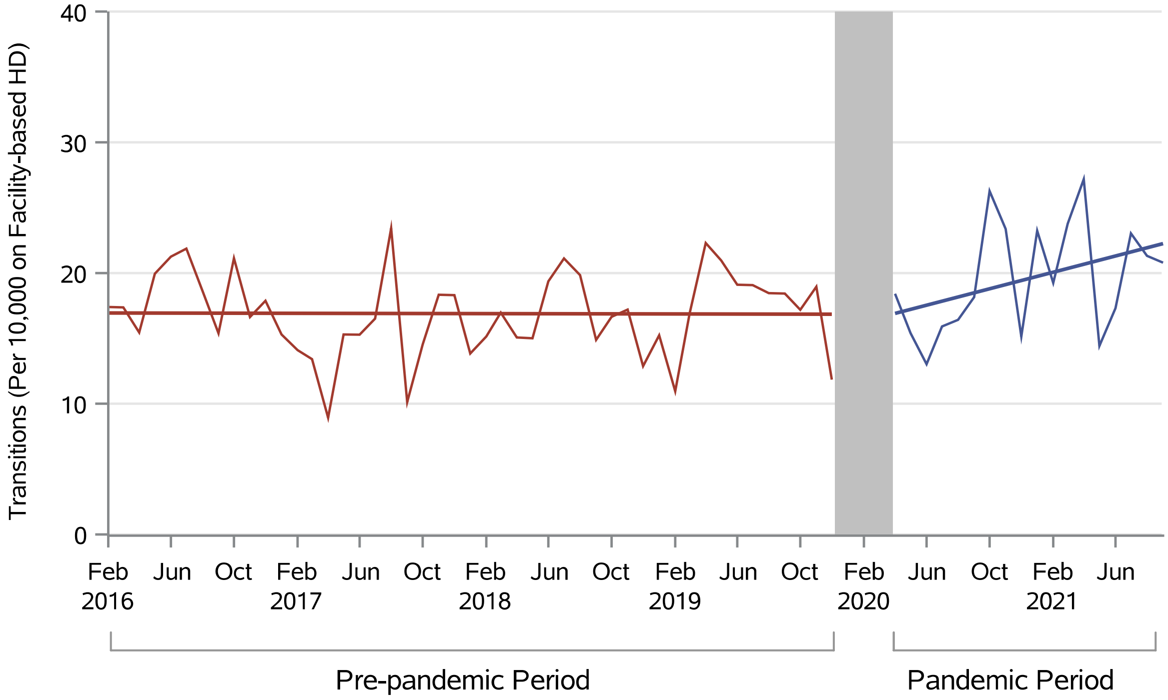

time per 10,000 monthly prevalent individuals on facility-based hemodialysis.

Figure S3. Transitions from peritoneal dialysis to facility-based hemodialysis in Canada over time per 10,000 monthly prevalent individuals on peritoneal dialysis.

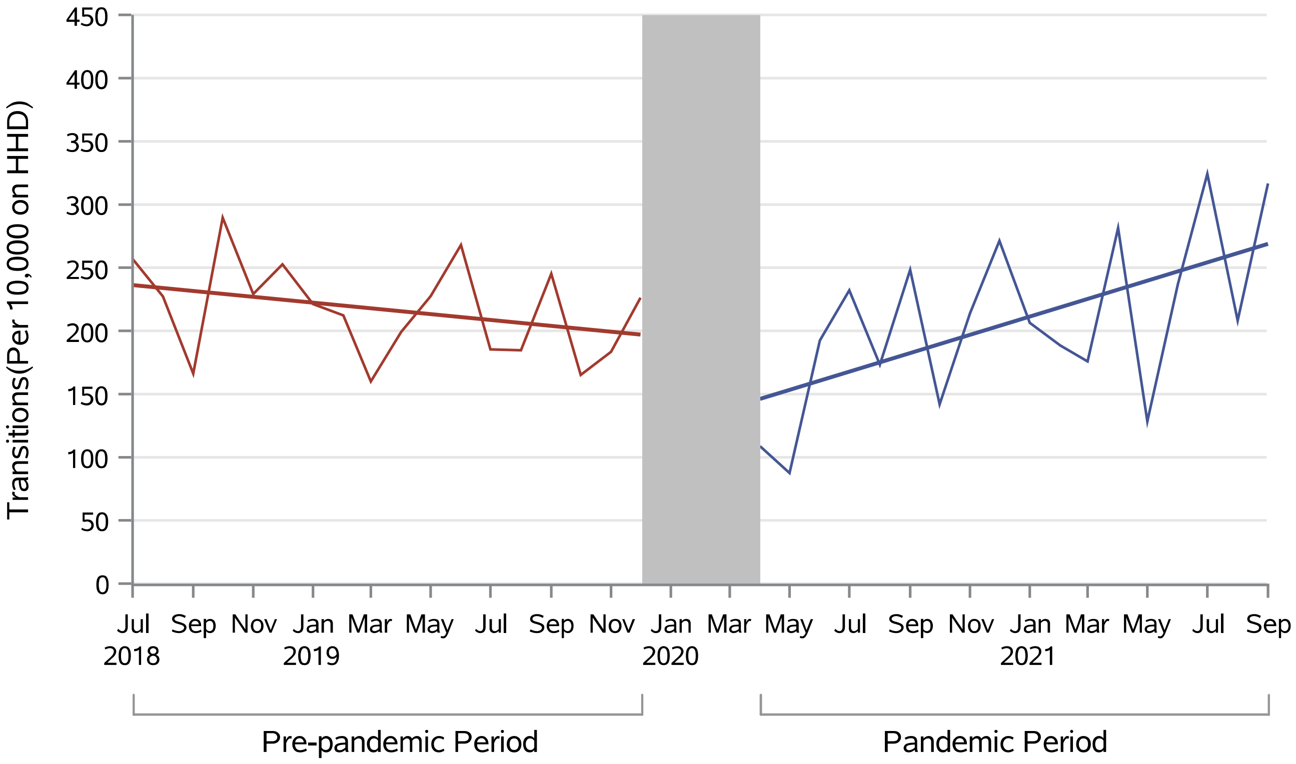

Figure S4. Transitions from home hemodialysis to facility-based hemodialysis in Canada over time per 10,000 monthly prevalent individuals on home hemodialysis.

Supplemental Table

Table S1. Comparison of average monthly transfers to facility over a year per 10,000 individuals on home dialysis when not including versus including individuals prevalent on PD for the study duration (2016-2021)

Year	Average monthly transfers to facility in a year	Average monthly prevalence of individuals on home dialysis (*excluding individuals on PD for duration of study*)	CIHI yearly prevalence of individuals on home dialysis (*complete*)[1]	Transfers to facility per 10,000 using our average monthly prevalence as denominator	Transfers to facility per 10,000 using CIHI official yearly prevalence as denominator
**2016**	33	1529	5425	217	61
**2017**	50	2169	5613	232	90
**2018**	69	2566	5707	269	121
**2019**	73	2697	5826	270	125
**2020**	78	2759	6090	281	127
**2021**	83	2662	6134	312	135

1. Canadian Institute for Health Information. Treatment of End-Stage Organ Failure in Canada CORR, 2012 to 2021: End-Stage Kidney Disease and Kidney Transplants — Data Tables. Ottawa, ON: CIHI; 2023.
